# Facilitators and barriers for the use of ultraviolet-C disinfection for patient room cleaning at VA hospitals: a qualitative analysis

**DOI:** 10.1017/ice.2025.10342

**Published:** 2026-02

**Authors:** Kimberly C Dukes, Stacey M. Hockett Sherlock, Cassie Cunningham Goedken, AM Racila, Julia Friberg Walhof, Daniel Suh, Michihiko Goto, Bernardino Guerrero, Trina Zabarsky, Eli N Perencevich

**Affiliations:** 1 Center for Access and Delivery Research and Evaluation, Iowa City Veterans Affairs Health Care System, Iowa City, IA, USA; 2 Division of General Internal Medicine, Department of Internal Medicine, Carver College of Medicine, University of Iowa, Iowa City, IA, USA; 3 Division of Infectious Diseases, Department of Internal Medicine, Carver College of Medicine, University of Iowa, Iowa City, IA, USA; 4 Environmental Programs Service (EPS), Veterans Affairs Central Office, Washington, DC, USA

## Abstract

To evaluate ultraviolet-C (UV-C) disinfection, we interviewed 34 personnel at 22 Veterans Affairs (VA) hospitals. Barriers included safety concerns, patient volumes, staffing, and costs. Facilitators included education and interprofessional communication. An implementation toolkit, interprofessional collaboration, and leadership support could optimize UV-C integration into VA infection prevention.

## Background

A hospital patient housed in a room where a previous patient had a multidrug-resistant organism (MDRO) has twice the risk of acquiring that MDRO.^1^ There are barriers to optimal environmental cleaning in hospitals, including for Environmental Management Service (EMS) staff,^2,3^ who often have limited training and high turnover, are not integrated into healthcare teams or empowered to change practices, and experience high time pressure to disinfect rooms quickly.

To improve disinfection, hospitals have added technologies like ultraviolet (UV-C) light devices that emit short-wavelength light. We previously found that UV-C implementation in Veterans Affairs (VA) hospitals was associated with a 19% reduction in gram-negative bloodstream infections (BSIs) but with significant hospital variation in BSI improvements.^4^ To investigate this variation, this project aimed to evaluate facilitators and barriers for UV-C disinfection as an adjunct to patient room disinfection across VA hospitals.

## Methods

### Data Collection

We invited personnel at 40 US VA hospitals that reported using UV-C disinfection during the 2010–2018 survey.^4^ From June 2022 to May 2023, we conducted nationwide interviews with hospital staff (EMS leaders, EMS personnel, infection preventionists, and multidrug-resistant-organism coordinators). The study was approved by the University of Iowa Institutional Review Board (#202112202) and the Research and Development Committee at the Iowa City VA. Prospectively developed interview guides included constructs of external and internal environments, organization, tools and technology, tasks, and persons. The qualitative team (KCD, SHS, CCG, AMR, JFW) conducted and recorded in-person or MS Teams interviews: (a) brief interviews focused on device types, types of rooms disinfected, and UV-C processes, and (b) in-depth individual or group interviews. Interviewers summarized brief interviews using a topical template; in-depth interviews were transcribed. We imported audited transcripts and template summaries into qualitative analysis software, MAXQda.^5^

### Data Analysis

We conducted template analysis of brief-interview summaries and consensus-based thematic analysis^6^ of in-depth interviews using an adapted codebook, adding emergent inductive themes and documenting changes systematically. After finalizing the codebook, we applied codes to template summaries.

## Results

Table 1 provides information about hospitals and participants. Thirty-four staff members participated. Table 2 provides exemplar quotes for selected themes.

**Table 1.** Characteristics of participating hospitals and hospital personnel

	*N*
**VA Hospitals contacted**	40
**VA Hospitals participating**	22 (55%)
*By census region:*	
Northeast	6
Midwest	7
South	8
West	1
**Hospital staff participating (no overlap in individual interviewees; multiple interviewees could participate at each hospital)**	34
Brief interview	20
In-depth interview	14

**Table 2.** Exemplar quotes for selected themes

Attitudes about whether UV-C works vary across and within hospitals	“[Non-EMS staff think it’s] a magic kill-everything machine.” [EMS leader, Site 8] “I was surprised in our VISN [Veterans Integrated Service Network] how many IPCs [Infection Prevention and Control] push back or don’t support UVC because they think it will give EMS a false sense of security.” (Infection Preventionist, Site 12) “[Previously] all we did was a two-step [disinfection process] and everything was good, right? The infection rate was still … low enough. There was no spike [in infections], so why is it [UV-C] becoming a requirement?” (EMS leader, Site 16) “We did have an increase in some infections when we stopped using UVC” (Infection Preventionist, Site 16)
Distrust and concerns about UV-C evidence	“I feel comfortable saying that…these machines aren’t doing what they say they do” (EMS leader 1, Site 13) “… validated data by the VA would be … a good thing…. You start researching these [other] researchers, well they work for [the manufacturer].” (EMS leader 2, Site 13) “I’m more of a naysayer… Everyone should know how and why it works and what it *doesn’t* do.” (MDRO Coordinator, Site 17)
Employees express concerns about safety	“…everyone was [saying] ‘Oh my God. Like, how is this gonna play out? Like, am I gonna put the little red goggles on, like I’m getting a tan?’” (EMS leader, Site 9) “[EMS staff] need to learn what the machines do, how they work, you don’t have to be afraid of it.” (EMS leader, Site 8) “The only problem I had with it was the smell and … if we could have something a little bit different to put on the doors to let people know that we were running it and not to open it. But you still have people still opening up the doors.” (EMS staff member, Site 13) “We had staff worries about ozone investigated…” (Infection Preventionist, Site 14)

### Summary of UV-C use

**Figure 1.**
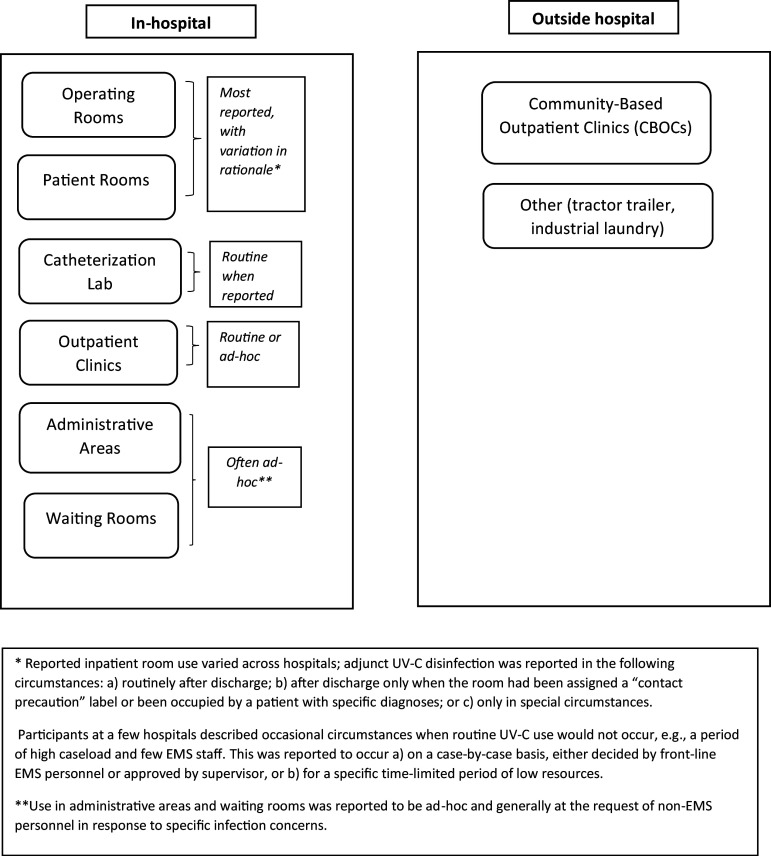
Reported UV-C use locations.

Twenty-one of 22 participating hospitals reported using UV-C disinfection in at least one hospital setting, with variation in locations, frequency, and rationales (Figure 1). One had discontinued all UV-C use. Another discontinued UV-C in patient rooms and reduced its use in operating rooms to after-hours, but continued its use in the catheterization lab and gastrointestinal clinic.

### Manual cleaning first

All interviewees emphasized that manual cleaning and disinfection was the priority, with UV-C as an adjunct process, sometimes described using metaphors like a “cherry on top” or “clear nail polish on top of a painted nail.” A frequently expressed concern was that EMS staff might overestimate the impact of UV-C disinfection and reduce their manual cleaning.

At one hospital with this concern (“Before, EMS and nurses used to think UV was the be-all end-all”), EMS began reporting both UV-C usage and microbiology testing results at that hospital, to clarify that manual cleaning remained vital (Site 18).

### Attitudes about UV-C

Attitudes were mixed regarding UV-C’s efficacy, feasibility, and acceptability. Many viewed UV-C as helpful for infection prevention (eg, “fantastic tool,” “I’m a big believer”) and multiple facilities reported successful implementation once procedures were in place and personnel were educated on the process and the technology.

However, sometimes leaders’ skepticism led to UV-C being discontinued or used inconsistently. Some personnel were not persuaded that UV-C was effective in killing organisms. Many distrusted manufacturer claims regarding exposure times and efficacy. For a few participants, even data from a clinical trial was insufficient evidence if it involved non-VA settings. A few participants did not perceive that UV-C reduced local infection rates.

Leadership support of UV-C, tied to their perception of its efficacy, affected implementation, including its discontinuation. At site 16, the EMS chief stopped using UV-C after previously working in 2 other VA hospitals that used it. At Site 4, two participants reported their EMS chief was “not a fan” and thought UV-C was ineffective; while this hospital had UV-C devices, implementation was “rocky at best” with staff “pushback,” frequent device breakage, and storage and access obstacles. At hospitals where leadership supported UV-C use (eg, “our chief is passionate”), implementation succeeded despite barriers (eg, time, storage) that deterred other hospitals.

Participants also described employee concerns about device safety, including smell, eye injuries, sunburns, and ozone exposure, especially early in implementation.

### Other barriers

Other barriers included high patient volumes requiring fast room turnovers, reduced EMS staffing, equipment and maintenance costs, technology variation, concerns about potential degradation of equipment and furnishings, effective testing, and competing priorities. Minor barriers included user error, inadequate storage space, bulb or device breakage, and device element malfunction or loss.

### Facilitators

#### COVID-19

Participants reported that COVID-19-related funds and leadership backing supported UV-C integration and pandemic-related infection concerns increased colleagues’ respect for EMS staff knowledge.

#### Role of education

Hospitals that reported successful UV-C integration emphasized training. Education frequency and types (hands-on, formal; manufacturer-led or internal) varied. Several hospitals conducted manufacturer-led training periodically and held refresher courses annually. However, several hospitals reported that they did not train all EMS employees to use UV-C, either because some employees were assigned to disinfect spaces where UV-C was not typically used, or some were uncomfortable with the technology. Education, when provided, reassured personnel about safety and improved employees’ knowledge and their skills, eg reducing bulb breakage.

#### Collaboration

Given concerns about room turnover and high census counts, EMS and nursing staff sometimes experienced frustration with UV-C’s addition, potentially exacerbating negative relationships between the two workforces. Participants reported that “relationship building” and improving communication among EMS, nursing, and infection control personnel could create a supportive environment.

For example, one EMS chief described a non-collegial situation when they assumed leadership. Within that context, “nursing would get mad that…we had to spend an extra 10 minutes cleaning a room [by using UV-C].” In response, the EMS leaders took steps to improve communication with nursing about EMS practices, including UV-C use. They “embedded” EMS staff in specific units to learn the nurses’ workflow and establish relationships, and educated nurses about EMS roles, responsibilities, and processes: “it took *us* learning about *their* jobs, *them* learning about *ours*.” These strategies allowed EMS and nursing staff to plan for both manual and UV-C disinfection during room turnovers and integrate this extra time into both EMS and nursing workflows.

At another hospital, IP personnel “micromanage[d]” environmental cleaning and UV-C use, eg. reading UV-C usage reports, conducting microbiology testing on all rooms to evaluate disinfection, then critiquing EMS. To reset relationships between IP and EMS, EMS took over control of testing and UV-C reports, and IP moved from “tattling” and “correcting” to acting as a “partner” that respected EMS’ professionalism.

#### Professionalism and commitment to infection prevention

Some participants at hospitals that successfully implemented UV-C into environmental cleaning described it as a way to show Veterans and families their commitment to patient safety or improve the hospital’s perceptions of EMS professionalism.

## Discussion

There is some evidence that UV-C can be an effective adjunct to environmental cleaning in reducing certain healthcare-associated infections in hospitals, and yet some caution exists.^7–9^ Since it is being used in the VA healthcare system, our qualitative study identified barriers and enablers of UV-C disinfection and variation in UV-C disinfection locations and rationales. Some hospitals reported that UV-C was well integrated into their routine practices. However, barriers were significant enough that one hospital discontinued its use entirely, and another stopped in patient rooms. Literature suggests US EMS workers experience challenges related to work pressure, low staffing, high turnover, or feelings of undervalue.^2,3,10^ Counterbalancing these, we found that EMS personnel were proud of their infection prevention expertise and that UV-C use could strengthen colleagues’ perception of EMS professionalism. However, we also found personnel questioned UV-C’s efficacy and identified other barriers including cost, efficiency, and safety concerns.

Goedken et al^3^ note the benefit of integrating EMS personnel into the larger healthcare team. We also found that greater collaboration among nursing, IP, and EMS personnel can help support EMS staff to integrate UV-C into routine environmental cleaning. Other facilitators included leadership support of UV-C use, personnel’s professional competence and commitment to infection prevention, and COVID-19-related financial resources and infection concerns.

These VA hospitals used a wide variety of UV-C devices and had varied UV-C locations, processes, and procedures. Thus, our findings may not be generalizable. However, findings were similar across hospitals, and many barriers and facilitators we identified may transfer to UV-C implementation in a range of hospitals.

## Conclusions

Given the potential benefits of UV-C, efforts to address barriers and support facilitators could drive implementation. A UV-C implementation toolkit, informed by VA staff feedback, may improve acceptability of UV-C technology among EMS staff. Interprofessional collaboration and leadership support is likely to enhance integration of UV-C into infection prevention systems in VA hospitals.
